# The influence of maturation and sex on intracranial blood velocities during exercise in children

**DOI:** 10.1152/japplphysiol.00478.2023

**Published:** 2023-12-21

**Authors:** Andrew J. M. Douglas, Jack S. Talbot, Dean Perkins, Tony G. Dawkins, Jon L. Oliver, Rhodri S. Lloyd, Philip N. Ainslie, Ali McManus, Christopher J. A. Pugh, Rachel N. Lord, Mike Stembridge

**Affiliations:** ^1^Cardiff School of Sport and Health Sciences, Cardiff Metropolitan University, Cardiff, United Kingdom; ^2^Centre for Health, Activity and Wellbeing Research, Cardiff Metropolitan University, Cardiff, United Kingdom; ^3^Youth Physical Development Centre, Cardiff Metropolitan University, Cardiff, United Kingdom; ^4^Centre for Heart, Lung, and Vascular Health, School of Health and Exercise Sciences, University of British Columbia, Kelowna, British Columbia, Canada; ^5^Department of Sport Science, University of Innsbruck, Innsbruck, Austria; ^6^Sports Performance Research Institute New Zealandy, AUT University, Auckland, New Zealand; ^7^Centre for Sport Science and Human Performance, Waikato Institute of Technology, Waikato, New Zealand

**Keywords:** adolescents, brain blood flow, cerebral blood flow, cerebral blood velocity, exercise, exercise in adolescents

## Abstract

Cerebral blood velocity (CBv) increases in response to moderate exercise in humans, but the magnitude of change is smaller in children compared with postpubertal adolescents and adults. Whether sex differences exist in the anterior or posterior CBv response to exercise across pubertal development remains to be determined. We assessed middle cerebral artery (MCAv) and posterior cerebral artery (PCAv) blood velocity via transcranial Doppler in 38 prepubertal (18 males) and 48 postpubertal (23 males) with cerebrovascular and cardiorespiratory measures compared at baseline and ventilatory threshold. At baseline, MCAv was higher in both sexes pre- versus postpuberty. Females demonstrated a greater MCAv (*P* < 0.001) than their male counterparts (prepubertal females; 78 ± 11 cm·s^−1^ vs. prepubertal males; 72 ± 8 cm·s^−1^, and postpubertal females; 68 ± 10 cm·s^−1^ vs. postpubertal males; 62 ± 7 cm·s^−1^). During exercise, MCAv remained higher in postpubertal females versus males (81 ± 15 cm·s^−1^ vs. 73 ± 11 cm·s^−1^), but there were no differences in prepuberty. The relative increase in PCAv was greater in post- versus prepubertal females (51 ± 9 cm·s^−1^ vs. 45 ± 11 cm·s^−1^; *P* = 0.032) but was similar in males and females. Our findings suggest that biological sex alters anterior cerebral blood velocities at rest in both pre- and postpubertal youth, but the response to submaximal exercise is only influenced by sex postpuberty.

**NEW & NOTEWORTHY** Cerebral blood velocity (CBv) in the anterior circulation was higher in females compared with males irrespective of maturational stage, but not in the posterior circulation. In response to exercise, females demonstrated a greater CBv compared with males, especially post-peak height velocity (post-PHV) where the CBv response to exercise was more pronounced. Our findings suggest that both CBv at rest and in response to acute submaximal exercise are altered by biological sex in a maturity-dependent manner.

## Graphical abstract

**Figure:**
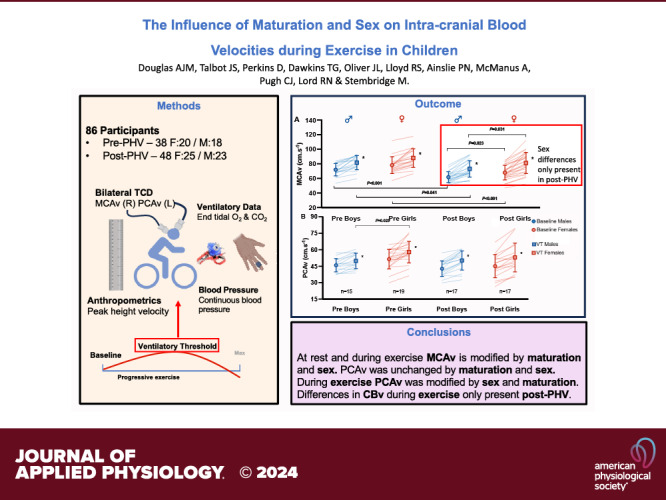

## INTRODUCTION

Cerebral blood flow (CBF) is modified by changes in arterial carbon dioxide (${\text{Pa}}_{{\text{CO}}_{2}}$), mean arterial pressure, cerebral metabolism, and autonomic nervous control (1). All of these are modified during exercise in an intensity-dependent manner (2). Changes in cerebral blood velocity (CBv) during exercise in adults can be broadly characterized by an increase in flow during mild to moderate exercise, before declining as maximal exercise intensity approaches (2) due to hyperventilation-induced hypocapnia (3). This pattern is seen in adults, adolescents, and children (4, 5); however, the relative increase in cerebral blood velocity (CBv) is greater in adults and adolescents compared with prepubertal children (5). The pubertal developmental window is associated with marked changes in stature, muscle mass, and metabolic demand. Such changes are partly driven by gonadal hormones that develop in a sex-specific manner and are known to influence cerebrovascular blood flow and function (6). However, whether pubertal development results in sexual dimorphism in regional CBv responses to exercise at the ventilatory threshold in both the anterior and posterior cerebral circulations remains to be explored.

Estrogen is a fundamental modulator for endothelium nitric oxide production (7), a key vasoactive metabolite released in response to increases in shear stress and pH (8). Although estrogen favors vasodilation, androgens such as testosterone increase vascular tone and suppress endothelium-dependent dilation (9–11). Sex-specific hormonal profiles exhibited by males and females during puberty are believed to underpin the divergent nonlinear trajectories of CBF at rest (12). Satterthwaite et al. (12) observed a continued decline in males from late childhood to adulthood and an increase from midpuberty in females, suggesting that the influence of sex-specific hormones is more apparent during the later years of puberty. Given the role sex hormones are likely to play in the regulation of CBF at rest, and particularly in response to changes in shear stress and ${\text{Pa}}_{{\text{CO}}_{2}}$, it is plausible that the trajectory of the CBF response to exercise in children is also sex specific. Two studies have examined the CBv response to exercise in children, adolescents, and adults and both found no sex differences; however, neither study was specifically designed to address sex differences (*n* = ∼7 in each sex) (4, 5). Although the CBv response to exercise does not appear to differ by sex in adults (4, 5), males achieve a greater cerebral perfusion pressure (13, 14). In adolescents, systolic blood pressure is higher in boys versus girls and in post- versus prepubertal children (15). However, whether maturation and sex interact to influence the CBv response to exercise remains to be determined. Moreover, studies examining differences in the CBv response to exercise have, to date, focused on the anterior circulation. Regional differences in cerebral vascular control exist between adolescent males and females (16) with a greater autoregulation, known as the ability to buffer changes in blood pressure, reported in the posterior circulation of females (17). Furthermore, it is currently unknown if the exercise-induced CBv response to exercise is regional specific, although adult data exist (18), the response in pediatrics and adolescents is underreported.

The aims of this study were to *1*) investigate whether sex differences are apparent in the CBv response to submaximal exercise in children and adolescents and *2*) explore the influence of maturation and sex on regional CBv distribution. In relation to these aims, we hypothesized that *1*) following puberty, females will demonstrate a greater CBv response to exercise compared with males, with no sex differences observed prepuberty and *2*) CBv to the posterior circulation will be similar between pre- and postpubertal children, but greater in females compared with males.

## METHODS

### Ethical Approval

All experimental protocols and procedures were approved by the Biomedical Sciences Research Ethics Committee at Cardiff Metropolitan University (PGR-1339), and conformed to the Declaration of Helsinki, except for registration in a database. All participants and parents/guardians were informed of the study design and methods verbally and in age-appropriate written information sheets before written informed consent and assent to participate were provided.

#### Participants.

Participants were recruited from local schools, sports clubs, and leisure activity groups. A total of 141 participants were recruited. To address our aims, participants were categorized by their estimated somatic maturation, using the Mirwald (19) equation of peak height velocity (PHV). Participants were classified as pre- or post-PHV, pre-PHV participants were at least 0.5 yr away PHV, and post-PHV participants were at least 0.5 yr post-PHV. The measurement of PHV has a standard error of 0.5 yr (19); as a result, participants who fell within −0.5 yr and 0.5 yr from their PHV were excluded from our analyses (*n* = 22). A further three participants were excluded due to insufficient transcranial Doppler ultrasound (TCD) signal at baseline, 24 participants were excluded based on TCD signal dropout due to excessive movement during the exercise test. Four participants were excluded for not achieving an age-appropriate respiratory exchange ratio (RER) at maximal exercise, an additional participant was excluded due to a response that was characterized as a significant outlier. A total of 86 participants were included in our analyses, with *n* = 18 pre-PHV males, *n* = 20 pre-PHV females, *n* = 23 post-PHV males, and *n* = 25 post-PHV females. Based on an a priori power calculation with a small effect size (*d* = 0.2) and 80% power, a total sample of *n* = 76 was required. We were unable to characterize sex hormones in these participants.

### Experimental Design

Participants visited the laboratory having refrained from caffeine for 12 h, fasted for 3 h, and abstained from strenuous activity for 12 h before visiting the laboratory. The visit began with anthropometric measures and baseline measures including height, sitting height, (SECA, 213, Germany), mass (SECA, 770, Germany), and brachial blood pressure using a size-specific automated blood pressure cuff (Omrom, M4, The Netherlands). Body composition was determined via skin fold assessment of the subscapular and triceps sites, with measurements taken on the right-hand side of all participants following the International Standards for Anthropometric Assessment guidelines (ISAK, 2001). Slaughter et al.’s (20) equation was used to calculate body fat percentage and lean body mass (20). CBv was assessed using TCD. Respiratory gas analyses were measured continuously at rest and throughout the maximal exercise test, and CBv data were time-aligned. A maximal exercise test on an upright cycle ergometer (Excalibur; Lode BV, Groningen, The Netherlands) was used to determine the ventilatory threshold.

### Experimental Measures

#### Cerebral blood velocities.

Middle cerebral artery velocity (MCAv) and posterior cerebral artery velocity (PCAv) were continuously measured using a 2 MHz TCD (Mode, Spencer Technologies, Seattle, WA). TCD probes were attached using a headband, fitted appropriately to insonate the MCA and PCA through the transtemporal window, as previously described by Willie et al. (21). MCA and PCA waveforms were identified by the location of TCD probes, with velocity and waveform used as confirmation for vessel isonation. TCD data were captured (PowerLab, 16/30; ADInstruments; Oxford, UK), and subsequently analyzed (LabChart, ADInstruments, v.7).

### Dynamic Cardiorespiratory Measures

End-tidal carbon dioxide ($PET_{\text{C}{\text{O}}_{2}}$), oxygen uptake (V̇o_2_), carbon dioxide production (V̇co_2_), minute ventilation (V̇e), and tidal volume (*T*_V_) were measured at baseline and continuously during exercise, using a breath-by-breath gas analyzer (Oxycon, Pro; Jaeger, Warwick, UK), via a Hans Rudolph facemask. The ventilatory threshold was identified using the V-slope method (22). Blood pressure (Finometer Pro, Finapres, The Netherlands) and heart rate (Polar Electro, RS4000; Kemple, Finland) were measured continuously across baseline and exercise. Data at baseline were taken as a final 30 s at the end of a 5-min resting period.

#### Exercise test.

During the exercise test, participants performed a 3-min warm-up at 10 W. Power was then increased depening on the participant’s stature and activity levels using wattage increments based on anthropometrics and volume of habitual exercise participation, see Table 1.

**Table 1.** Exercise intensity increments

Volume of exercise participation	Height, cm	Intensity Increment, W/min
<3 h of exercise per week	110–124.9	10
	125–149.9	15
	>150	20
>3 h of exercise a week	110–124.9	15
	125–149.9	20
	>150	25

Participants were asked to maintain a cadence between 75 and 85 rpm for the duration of the test, to standardize resistance between groups, until a drop in cadence >5 rpm for more than 5 consecutive seconds against the increasing resistance (23) despite strong verbal encouragement. Maximal effort was confirmed with physiological criteria including breathing rigorously, sweating, an age-appropriate RER for maximal exercise (24), and using the Tanaka equation within 10 beats of age-predicted maximal heart rate (25).

### Data Processing

Baseline measures of CBv, ventilatory, and blood pressure data were taken in final 30 s of a 5-min resting period, during which participants were asked to sit quietly on the upright cycle ergometer. Measures at ventilatory threshold were taken at the 30-s preceding ventilatory threshold and averaged across this period. Given our aim was to identify the effect of sex on the CBv response to exercise, we chose a metabolic anchor that would result in the greatest change (4, 5). As such, we focused our data collection efforts on obtaining an optimal TCD signal up to this submaximal exercise intensity, and thereafter encouraged the participants to provide maximal effort to achieve a valid measure of cardiorespiratory fitness.

### Statistical Analysis

Descriptive data are expressed as means ± standard deviation. Baseline participant characteristics were compared using a mixed-model ANOVA with between-subjects effects for sex and maturation, and within-subjects effect of rest versus exercise-labeled time. The cerebrovascular and ventilatory response to exercise was tested for normality using Shapiro–Wilk and fit within normal distribution. Cerebrovascular data were assessed for outliers using the Grubbs’ outlier statistical test (26), α was set at 0.05. This identified one participant with a relative MCAv response of −22.6%. The absolute and relative MCAv data of this participant were removed from our analysis. Data were analyzed as absolute change from baseline to the ventilatory threshold, using mixed-model three-way ANOVAs to determine the main effects of sex, maturation, and exercise intensity and any interactions. Relative change was expressed as a percentage from baseline to the ventilatory threshold, was assessed using a two-way ANOVA by sex and maturation. Ventilatory threshold was chosen as the metabolic anchor at which the greatest change in CBv has been reported previously (4, 5). Where significant main or interaction effects existed, post hoc pairwise tests were conducted using Bonferroni correction with corrected *P* values reported. All statistical analysis was performed using SPSS (v.27, SPSS, Chicago, IL) with α set at 0.05. As recommended across pediatric exercise testing, data should be reported relative to lean body mass and scaled appropriately (27, 28). Values were allometrically scaled using a scaling exponent generated from lean body mass (LBM) data from the current study (LBM_1.065_) (27). Allometric scaling was used for the comparison of oxygen uptake at ventilatory threshold (VT) and maximum (Table 2). 

**Table 2.** Participant characteristics at rest and response to incremental exercise

	Pre Males	Pre Females	Post Males	Post Females	Maturation (*P* Value)	Sex (*P* Value)	Interaction (*P* Value)
*n*	18	21	23	25			
Height, cm	147 ± 9*⊥	137 ± 9*	178 ± 9⊥	164 ± 7	***P* = <0.001**	***P* = <0.001**	*P* = 0.232
Weight, cm	38.2 ± 7.2⊥	31.6 ± 5.9*	64.0 ± 10.1⊥	54.6 ± 9.1	***P* = <0.001**	***P* = <0.001**	*P* = 0.449
Age, yr	11.1 ± 1.6*⊥	10.0 ± 1.4*	16.0 ± 1.0⊥	14.0 ± 1.5	***P* = <0.001**	***P* = <0.001**	*P* = 0.126
PHV, yr	−2.4 ± 1.0*	−1.9 ± 1.0*	2.1 ± 0.8	1.9 ± 1.0	***P* = <0.001**	*P* = 0.580	*P* = 0.067
LBM, kg	30.5 ± 5.3*⊥	25.6 ± 3.9*	54.5 ± 6.4⊥	42.4 ± 6.4	***P* = <0.001**	***P* = <0.001**	*P* = 0.005
BMI	17.49 ± 2.22	16.57 ± 1.85	20.08 ± 2.36	20.23 ± 2.74	***P* = <0.001**	***P* = <0.001**	*P* = 0.005
BL MAP, mmHg	76 ± 5*	75 ± 7	81 ± 6	78 ± 6	***P* = <0.001**	*P* = 0.110	*P* = 0.417
BL SBP, mmHg	105 ± 6*	108 ± 8*	118 ± 7⊥	110 ± 8	***P* = <0.001**	***P* = <0.001**	*P* = 0.158
BL DBP, mmHg	62 ± 6	62 ± 7	63 ± 6	62 ± 6	*P* = 0.950	*P* = 0.910	*P* = 0.744
BL heart rate, beats/min	62 ± 3	67 ± 4	63 ± 6	65 ± 4	*P* = 0.725	*P* = 0.003	*P* = 0.130
VT (V̇o_2_ L·kg·min^1.065^)	26.4 ± 8.0	24.2 ± 5.4	25.1 ± 8.2	23.1 ± 6.7	*P* = 0.333	*P* = 0.173	*P* = 0.922
VT RER	0.89 ± 0.07	0.91 ± 0.08	0.93 ± 0.07	0.88 ± 0.06	*P* = 0.705	*P* = 0.193	*P* = 0.037
VT power, W	90 ± 45*	57 ± 30*	153 ± 77⊥	120 ± 69	***P* = <0.001**	*P* = 0.020	*P* = 0.717
Peak power, W	150 ± 50*	111 ± 40*	274 ± 77⊥	199 ± 75	***P* = <0.001**	***P* = <0.001**	*P* = 0.115

Data are presented as means ± standard deviation. Participant’s characteristics were compared using a two-way analysis of variance (ANOVA) with main effects for sex, maturation, and a sex, maturation interaction. Significance is represented in bold. BL, baseline; BMI, body mass index; DBP, diastolic blood pressure; LBM, lean body mass; MAP, mean arterial pressure; PHV, peak height velocity; RER, respiratory exchange ratio; SBP, systolic blood pressure; VT, ventilatory threshold.

*A significant maturation differences within sex; ⊥a significant sex difference within maturation.

## RESULTS

### Influence of Maturation and Sex on Cardiorespiratory and Cerebrovascular Measures at Rest

As expected, height and weight were significantly greater in post-PHV compared with pre-PHV participants (Table 2). Male and female groups were closely matched for PHV (*P* = 0.58), although females were older in chronological age (*P* < 0.001). Mean arterial and systolic blood pressures were higher in post- versus pre-PHV groups (*P* = 0.005; Table 2), and were higher in males compared with females (*P* < 0.001; Table 2). Diastolic blood pressure was not different with maturation (*P* = 0.950) or sex (*P* = 0.910; Table 2).

**Figure 1.**
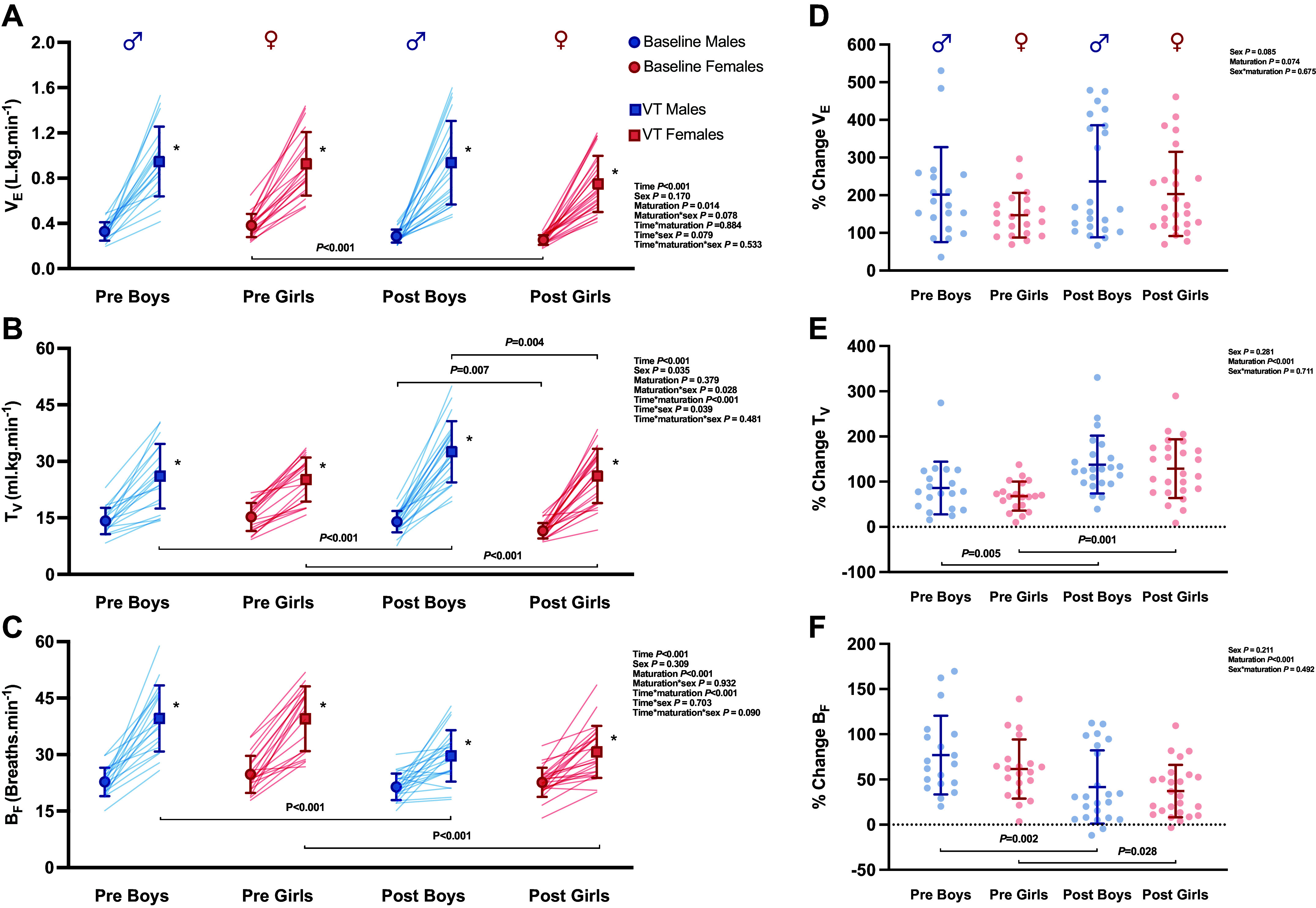
Cardiorespiratory response at baseline depicted in circles and in response to exercise at the ventilatory threshold depicted in squares (*A*–*C*), and the percentage change from baseline to ventilatory threshold (*D*–*F*). Symbols represent individual data points; significance is represented with *P* value between groups. *Pairwise difference between baseline and ventilatory threshold. B_F_, breathing frequency; T_V_, tidal volume; V̇e, minute ventilation.

**Figure 2.**
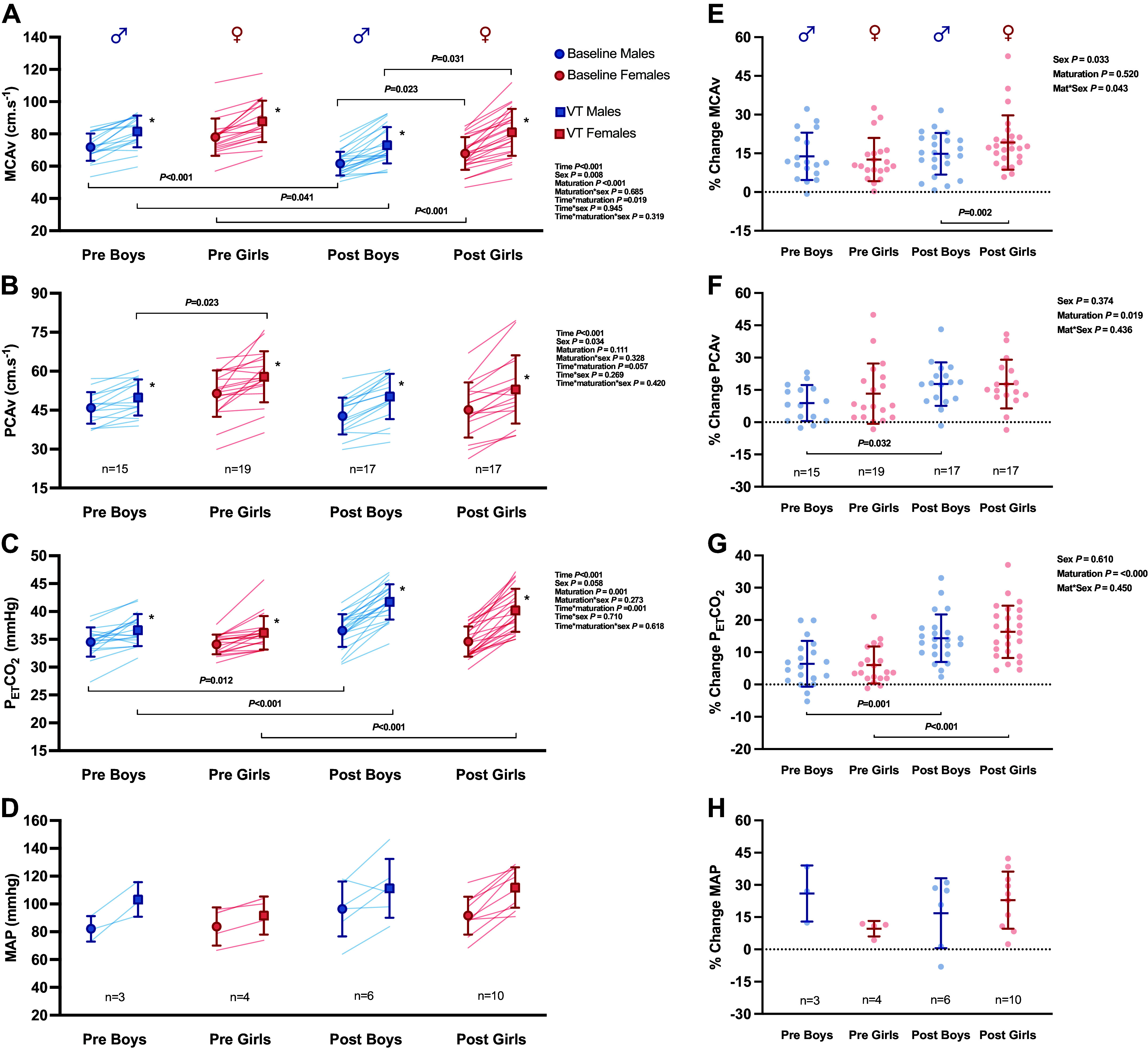
Individual and mean cerebrovascular and cardiorespiratory response at baseline depicted in circles and in response to exercise at ventilatory threshold depicted in squares (*A*–*D*), and the percentage change from rest to ventilatory threshold (*E*–*H*). *Pairwise difference between baseline and exercise at ventilatory threshold. MAP, mean arterial pressure; MCAv, middle cerebral artery velocity; PCAv, posterior cerebral artery velocity; $P\text{E}{\text{T}}_{\text{C}{\text{O}}_{2}}$, partial pressure end-tidal carbon dioxide.

Baseline V̇e relative to body mass was not different with maturation, although breathing frequency (B_F_) was greater in both pre-PHV males and females compared with their post-PHV sex-matched counterparts (Fig. 1, *A* and *C*). $P\text{E}{\text{T}}_{\text{C}{\text{O}}_{2}}$ was higher in pre- (34.5 mmHg) versus post-PHV (36.6 mmHg) males at baseline (*P* = 0.012; Fig. 1), but no differences were present in females (*P* = 0.489; Fig. 2*C*). Furthermore, *T*_V_ relative to body weight was greater in post-PHV males compared with post-PHV females (*P* = 0.004; Fig. 1*D*). There were no sex differences in $P\text{E}{\text{T}}_{\text{C}{\text{O}}_{2}}$ (Fig. 2*C*).

Baseline absolute MCAv was higher in pre- versus post-PHV males and females (*P* < 0.001), with both pre-PHV males and females displaying a greater absolute MCAv than their sex-matched post-PHV counterparts (*P* < 0.001 and *P* < 0.001), respectively. Baseline absolute MCAV was greater in females compared with males (*P* = 0.008), baseline MCAv was greater in post-PHV females compared with post-PHV males (*P* = 0.031; Fig. 2*A*) with no effect of sex between pre-PHV males and females. Baseline absolute PCAv was greater in females compared with males (*P* = 0.034; Fig. 2*B*).

### Influence of Maturation and Sex on the Cardiorespiratory and Cerebrovascular Response to Exercise

Power at ventilatory threshold was greater in post- versus pre-PHV (*P* < 0.001) and in males versus females (*P* = 0.020). There was no main effect of sex or maturation on maximal heart rate. Maximal oxygen consumption was comparable between pre- and post-PHV children, but higher in males (Table 2). However, V̇o_2_ at ventilatory threshold was not different by maturation or sex.

As expected during exercise, *T*_V_, B_F_, and V̇e increased in all groups (*P* < 0.001). *T*_V_ was higher in post-PH_V_ males and females compared with pre-PHV counterparts (Fig. 1*B*). In response to exercise, $P\text{E}{\text{T}}_{\text{C}{\text{O}}_{2}}$increased from baseline to ventilatory threshold (*P* < 0.001) in all groups and was greater in post-PHV females and males compared with their pre-PHV sex-matched counterparts (both *P* < 0.001; Fig. 1*C*). Likewise, %Δ$P\text{E}{\text{T}}_{\text{C}{\text{O}}_{2}}$ was greater in post-PHV males and females compared with their sex-matched pre-PHV counterparts (*P* < 0.001; Fig. 2*G*). Neither $P\text{E}{\text{T}}_{\text{C}{\text{O}}_{2}}$ nor %Δ$P\text{E}{\text{T}}_{\text{C}{\text{O}}_{2}}$ differed by sex at ventilatory threshold.

Absolute MCAv was greater at ventilatory threshold versus baseline across all groups (*P* < 0.001). Consistent with baseline, a main effect of maturation in absolute MCAv was present at ventilatory threshold. In response to exercise, absolute MCAv at ventilatory threshold was greater in post-PHV females (*P* < 0.031; Fig. 2*A*) compared with post-PHV males, with no differences observed in pre-PHV males and females.

%ΔMCAv at ventilatory threshold was greater in females compared with males (*P* = 0.033; Fig. 2*E*), more specifically post-PHV females had a greater relative increase in MCAv (*P* = 0.002) compared with post-PHV males, with no differences in pre-PHV. $P\text{E}{\text{T}}_{\text{C}{\text{O}}_{2}}$ was similar between sexes at ventilatory threshold, but greater in post-PHV compared with pre-PHV participants. In response to exercise, $P\text{E}{\text{T}}_{\text{C}{\text{O}}_{2}}$ was greater in post-PHV females compared with pre-PHV females, with no differences in males.

In the posterior circulation, absolute PCAv increased from baseline to ventilatory threshold across all groups (*P* < 0.001). There was no effect of maturation on absolute PCAv at ventilatory threshold (*P* = 0.111; Fig. 2*B*); however, when expressed as %ΔPCAv, a main effect of maturation was observed (*P* = 0.019; Fig. 2*F*), with a greater response to exercise in post-PHV males compared with pre-PHV males, with no difference of maturation observed in females. Moreover, there was a main effect of sex, with a greater absolute PCAv at ventilatory threshold in pre-PHV females versus males that were not present at rest (Fig. 2*B*). In response to exercise, mean arterial pressure (MAP) (Fig. 2, *D* and *H*) increased from rest; however, due to the small sample size, we are not able to draw statistical conclusions.

## DISCUSSION

In relation to our hypotheses, we accept our primary hypothesis that following puberty, females will demonstrate a greater CBv response to exercise compared with males, with no sex differences observed prepuberty. The primary findings of the current study are: *1*) absolute MCAv at baseline was greater in females compared with males post-PHV, with no differences pre-PHV; *2*) in response to exercise, MCAv was higher in post-PHV females compared with post-PHV males; however, no absolute MCAv sex differences were observed in pre-PHV groups, and *3*) in the posterior circulation, absolute PCAv was greater in pre-PHV females versus males with no differences post-PHV at ventilatory threshold. The current findings highlight sexual dimorphism in the cerebral circulation at rest and in response to exercise that is across somatic maturity.

### Higher Regional Cerebral Blood Velocity in Females versus Males at Rest

In the present study, females displayed a significantly greater MCAv at rest compared with their male counterparts post-PHV. At ventilatory threshold, post-PHV females had a greater absolute MCAv compared with post-PHV males, with no differences observed between pre-PHV males and females. Relative MCAv was significantly greater in females compared with males, specifically, post-PHV females had a greater exercise response compared with post-PHV males. There was no main effect of sex in the relative PCAv change from baseline to VT. A higher resting MCAv has been reported previously in healthy adult females using TCD (28) and in children as young as 10 yr old (29). A larger study using magnetic resonance imaging demonstrated a similar global CBF between males and females during early puberty, followed by an upward trajectory in females at midpuberty, whereas CBF declined in males from early puberty to late (12). In the posterior circulation, we observed no differences in PCAv at rest between pre- versus post-PHV groups. In contrast, previous work demonstrated PCAv derived from MRI to be greater in children compared with adults (30). Despite differences in methodology, the majority of studies demonstrate a higher cerebral blood flow and velocity in females compared with males (12), although this is not always the case (30, 31). At least in the upright posture, our data confirm that MCAv is higher in females versus males irrespective of maturational stage. The mechanism driving the higher CBv in females remains poorly understood. Previously, sex differences in cerebral blood flow have been attributed to circulating hormones (6), with both estrogen and testosterone acting via modulation of endothelial factors, such as nitric oxide availability, prostaglandins, and endothelial-derived hyperpolarizing factor (6). Given that CBv is elevated in prepubertal youth (4, 5) where hormonal sex differences are far less pronounced (32) it is plausible that the effect of sex hormones would be dampened during this phase compared with later in pubertal development. However, the present study did not measure circulating hormones and therefore cannot confirm the role of sex hormones on CBv at rest or during exercise. Indeed, females aged from 10 to 16 yr old report a higher MCAv and basilar artery velocity, compared with age-matched male counterparts (16). Other mechanisms are likely responsible for the differences between males and females at this age, including anatomical development (33), cerebral metabolism (34), and lifestyle factors such as exercise (35); however, we are yet to understand the effect of these variables on CBv in a longitudinal manner.

### Influence of Maturational Stage on Cerebral Blood Velocity during Exercise

In the present study, the relative increase in MCAv in response to exercise was greater in post- compared with pre-PHV, consistent with previous findings (4, 5). Previously, differences in MCAv response to exercise have been explained through the absence of a relationship between cerebral velocity and $P\text{E}{\text{T}}_{\text{C}{\text{O}}_{2}}$ in children compared with adults, during moderate exercise (4, 5). The assertion was originally based on a blunted response to changes in arterial blood gases in children (36). However, recent evidence of comparable cerebrovascular reactivity to carbon dioxide in children versus adults, albeit with a slower rate of change in children (37, 38) indicates that sensitivity to carbon dioxide does not explain the different trajectories in response to exercise. Moreover, the idea that age-related differences in CBv are driven by carbon dioxide is further challenged by the use of $P\text{E}{\text{T}}_{\text{C}{\text{O}}_{2}}$ as a surrogate for ${\text{Pa}}_{{\text{CO}}_{2}}$ (39–41). At rest, $P\text{E}{\text{T}}_{\text{C}{\text{O}}_{2}}$ provides a good index of ${\text{Pa}}_{{\text{CO}}_{2}}$ (42). However, in response to exercise the $P\text{E}{\text{T}}_{\text{C}{\text{O}}_{2}}$-${\text{Pa}}_{{\text{CO}}_{2}}$ gradient widens, and to a greater extent in adults versus children (43). It is generally accepted that ${\text{Pa}}_{{\text{CO}}_{2}}$ remains unaltered up to anaerobic threshold during exercise (44), although one study did report an increase in ${\text{Pa}}_{{\text{CO}}_{2}}$ in adolescents aged 14–21 yr at anaerobic threshold but no change in younger children (aged 8–11 yr). Without arterial blood gases in children, an obvious and significant ethical barrier, we cannot definitively conclude whether age-related differences in CBv are due to differences in ${\text{Pa}}_{{\text{CO}}_{2}}$.

A blunted CBv response to exercise in children (4) and adolescents (5) compared with adults may represent a limited blood flow reserve as a protective mechanism to avoid overperfusion (4) given the elevated CBv in children and adolescents compared with adults at rest (5). In response to exercise, systolic blood pressure increases, whereas diastolic blood pressure remains relatively unchanged (45). During exercise in children, Ellis et al. (4) observed no differences in MAP in response to exercise between children and adults. However, similar to the present study, Ellis et al.’s observations were limited to a small sample size. Larger studies have shown that systolic blood pressure increased more in postpubertal participants compared with their prepubertal counterparts, and was also greater in males compared with females (46); however, the sex difference was related to the greater absolute workload achieved in males. In the present study, we are limited to a subcohort due to excessive movement during exercise rendering the blood pressure signal unusable. As expected in response to continuous exercise, MAP displays a positive trend from rest to ventilatory threshold across all groups. Future research should aim to identify the blood pressure response in children across a range of exercise intensities and modalities.

### Sex Differences in the Cerebral Blood Velocity Response to Exercise

Post-PHV females demonstrated a higher absolute MCAv compared with post-PHV males, and a greater increase in response to exercise than their pre-PHV counterparts. The greater CBv during exercise in post-PHV females may be mediated by the divergence sex-specific hormone, with estrogen exerting a vasodilatory effect (47) on the smaller cerebral vessels (46). A direct effect of sex hormones needs to be confirmed through hormone measurement, indeed a limitation of the present study. Alternatively, mechanistically manipulating hormone levels with acute interventions (48) would account for individual differences in regulating hormone levels. Although, careful consideration of the ethical considerations of working with a young population is required. Although understanding the effect of circulating hormones on the CBv response to exercise is pertinent, other factors affecting CBv control during exercise warrant consideration. Recent findings by Tallon et al. (37) demonstrated the disparity between the mechanisms responsible for cerebrovascular control between children and adults in response to a fixed hypercapnic stimulus. Children displayed a slower time constant compared with adults, despite a similar magnitude of MCAv change. One mechanism thought to contribute toward the differences between children and adults is vasomotion, i.e., the change in blood vessel diameter (37). Vasomotion serves to protect the cerebral blood vessels by regulating velocity and pulsatility (49). This protective mechanism is thought to adapt with increasing maturation, and therefore may affect the vasomotion of cerebral blood vessels in response to dynamic hypercapnic challenges such as exercise. On the contrary, during high-intensity exercise posterior flow continues to increase above 80% of maximal work rate in adults compared with a decrease in anterior flow (3), although the present study exclusively measured CBv at baseline and ventilatory threshold, absolute PCAv was greater at ventilatory threshold compared with baseline across all groups, notwithstanding the increase in global flow in response to exercise, the increase in absolute PCAv might suggest the start of a regional-dependent distribution in flow, acting as a protective mechanism for overperfusion, and speculated to be a result of hyperventilatory educed hypercapnia (3). Future research should aim to understand the relationship between regional CBv distribution between sexes across maturation.

### Study Considerations/Limitations

TCD is limited to blood velocity as an index of CBF, with the underlying assumption that MCA and PCA vessel diameter is unchanged (50). However, it is currently the only method for insonation of the smaller cerebral vessels during upright dynamic exercise. We accept the limitations of TCD and interpret results accordingly. The data presented are cross-sectional in nature; to truly understand the changes from pre- to post-PHV in males and females and the influence of puberty, future research should aim to assess longitudinal changes across the pubertal spectrum. In the present study, we were unable to measure sex hormones, future work should aim to measure sex-specific hormones appropriately, with specific attention to the menstrual. Furthermore, investigating the subsequent effect on CBF during natural or induced fluctuations in hormone levels will provide a greater understanding of the role of sex-specific hormones on CBF across maturation.

### Translational Perspective

Advancing our understanding of cerebral blood flow regulation during normal healthy development will facilitate diagnosis, management, and treatment of a range of developmental and neurocognitive conditions experienced during childhood. For example, neuroimaging techniques have been used in the diagnosis and medical care of young athletes following concussion. To do so, it is vital we understand what is “normal” for cerebral structure and function, but considerable heterogeneity of neuroimaging measures have been reported in children following traumatic brain injury (51). We have found biological sex to influence cerebrovascular function, highlighting that it should be considered as our understanding of conditions such as pediatric concussion evolves. Moreover, adolescence is also a time period where sex differences manifest, not only in our physiology but in our physical activity levels. For example, the decline in activity across secondary school in girls is 7.4% compared with only 2.7% in boys (52). However, a historical bias toward the use of young males in biomedical research means there is a lack of high-quality and compelling evidence regarding the fundamental changes during maturation and the impact of exercise on female brain development. Females are often excluded based on the argument that hormonal changes increased variability within data sets, requiring a larger sample to achieve an adequate signal-noise ratio. It is precisely this hormone-related variability that we need to understand, if we are to promote healthy lifestyles for both sexes in childhood for continued benefit throughout the lifespan. Understanding how normal changes in sex hormones influence cerebral development will also provide a basis for understanding the pathophysiology of disorders associated with endocrine dysregulation such as polycystic ovary syndrome.

### Conclusions

Sex differences exist in the anterior and posterior circulations, these differences are dependent on maturity and region at rest and during exercise. Baseline blood velocity in the anterior cerebral circulation was similar between pre-PHV males and females. However, after the onset of puberty, post-PHV females displayed a greater baseline MCAv compared with maturation-matched males. Furthermore, in response to exercise, sex differences were present in post-PHV but not pre-PHV. Whereas in the posterior circulation, we observed no baseline differences in PCAv, in response to exercise pre-PHV females had a greater PCA velocity response compared with maturation-matched males. The differences highlighted in the present study identify the importance of understanding and accounting for maturational and sex-dependent cerebrovascular differences at rest and in responses to exercise.

## DATA AVAILABILITY

The data that support the findings of this study are openly available on Figshare at DOI: https://doi.org/10.6084/m9.figshare.24834684.

## GRANTS

M.S. and J.S.T. were supported by The Waterloo Foundation.

## DISCLOSURES

No conflicts of interest, financial or otherwise, are declared by the authors.

## AUTHOR CONTRIBUTIONS

J.S.T., D.P., T.G.D., and C.J.A.P., R.N.L., and M.S. conceived and designed research; J.S.T., D.P., T.G.D., and R.N.L. performed experiments; A.J.M.D., J.S.T., and A.M. analyzed data; A.J.M.D., J.S.T., A.M., C.J.A.P., R.N.L., and M.S. interpreted results of experiments; A.J.M.D. and J.S.T. prepared figures; A.J.M.D., J.S.T., and M.S. drafted manuscript; A.J.M.D., J.S.T., D.P., T.G.D., J.L.O., R.S.L., P.N.A., A.M., C.J.A.P., R.N.L., and M.S. edited and revised manuscript; A.J.M.D., J.S.T., D.P., T.G.D., J.L.O., R.S.L., P.N.A., A.M., C.J.A.P., R.N.L., and M.S. approved final version of manuscript.
